# A CSI-Based Indoor Positioning System Using Single UWB Ranging Correction

**DOI:** 10.3390/s21196447

**Published:** 2021-09-27

**Authors:** Keliu Long, Darryl Franck Nsalo Kong, Kun Zhang, Chuan Tian, Chong Shen

**Affiliations:** 1 State Key Laboratory of Marine Resources Utilization in South China Sea, School of Information and Communication Engineering, Hainan University, Haikou 570228, China; keliulong@hainanu.edu.cn (K.L.); darrylfranck@hainanu.edu.cn (D.F.N.K.); 2 School of Information and Communication Engineering, Hainan University, Haikou 570228, China; 3 Education Center of MTA, Hainan Tropical Ocean University, Sanya 572022, China; 4 Institute of Deep-Sea Science and Engineering, Chinese Academy of Sciences, Sanya 572000, China; tianc@idsse.ac.cn

**Keywords:** channel state information, deep learning, indoor localization, localization calibration, UWB ranging

## Abstract

A fingerprint-based localization system is an economic way to solve an indoor positioning problem. However, the traditional off-line fingerprint collection stage is a time-consuming and laborious process which limits the use of fingerprint-based localization systems. In this paper, based on ubiquitous Wireless Fidelity (Wi-Fi) equipment and a low-cost Ultra-Wideband (UWB) ranging system (with only one UWB anchor), a ready-to-use indoor localization system is proposed to realize long-term and high-accuracy indoor positioning. More specifically, in this system, it is divided into two stages: (1) an initial stage, and (2) a positioning stage. In the initial stage, an Inertial Measure Unit (IMU) is used to calculate the position using Pedestrian Dead Reckon (PDR) algorithm within a preset number of steps, and the location-related fingerprints are collected to train a Convolutional Neural Network (CNN) regression model; simultaneously, in order to make the UWB ranging system adapt to the Non-Line-of-Sight (NLoS) environment, the increments of acceleration and angular velocity in IMU and the increments of single UWB ranging measures are correlated to pre-train a Supported Vector Regression (SVR). After reaching the threshold of time or step number, the system is changed into a positioning stage, and the CNN predicts the position calibrated by corrected UWB ranging. At last, a series of practical experiments are conducted in the real environment; the experiment results show that, due to the corrected UWB ranging measures calibrating the CNN parameters in every positioning period, this system has stable localization results in a comparative long-term range. Additionally, it has the advantages of stability, low cost, anti-noise, etc.

## 1. Introduction

With the development of the Internet of Things (IoT), indoor Location Based Service (LBS) has aroused extensive research in recent years [1]. Though Global Navigation System (GNS), including Chinese BeiDou Navigation System (BDS) and American Global Positioning System (GPS), can provide high-accuracy positioning results in open-air scenarios [2], they are limited in indoor environments such as basements, tunnels, and even high-density building areas, due to attenuation and distortion of electronic signals on the surface of blocking objects [3]. Therefore, many positioning technologies have been proposed or improved to satisfy various indoor localization requirements including technologies such as Wi-Fi [4], UWB [5], Bluetooth [6], Zigbee [7], Ultrasonic [8], and more [9]. Broadly, current indoor positioning technologies can be classified into two types: fingerprinting-based methods and ranging-based methods.

In fingerprinting-based methods, the Wi-Fi-based positioning system is the most popular research field [10]. Wi-Fi sending equipment is widely deployed around us, thus, Wi-Fi signals are easy to use as position-related signals. However, Wi-Fi based indoor localization systems are sensitive to environment state change. With the rapid development of the Internet of Things (IoT), most indoor intelligent devices provide services based on high-accuracy positioning such as shopping navigation, elderly care services, smart building/city, prison security, smart manufacturing facility [11], etc. In order to improve the localization performance of Wi-Fi, most of the positioning schemes applied in a Wi-Fi-based system are fingerprint matching, which is divided into two stages: an off-line stage and an on-line stage. In the off-line stage, position-relative signal characteristics on different points are collected to construct a fingerprint database; the accuracy of the positioning result depends on the density of fingerprint collecting points, i.e., the higher density, the better result. In the on-line stage, the signal characteristics of unknown points are compared with fingerprints in the database using different algorithms, namely, K-Nearest Neighbor (KNN) [12], Weighted K-Nearest Neighbor (WKNN) [13], etc. The Wi-Fi-based positioning system is low-cost but laborious. Although it can reach a high accuracy in a short time; the accuracy will gradually degrade with time (as the environmental layout changes or due to pedestrian interference). Thus, the fingerprint database must be refreshed frequently, which limits the development of a fingerprint-based localization system. Zheng et al. [14] proposed a deep learning-based fingerprint updating scheme to alleviate the database refreshing issue. They used an autoencoder to extract current environmental characteristics using amplitude and phase of signal under unsupervised learning in the off-line stage, after which the trained autoencoders calibrated a real-time fingerprint in the on-line stage. It can work well under slight environmental change, however, as the author stated, the autoencoder-based fingerprint calibrated system is limited in a frequently changing environment. Huang et al. [15] adopted a marginalized particle extended Gaussian process (MPEG) to recursively refresh the fingerprint map, and pedestrian dead reckoning (PDR) is used to calibrate the location labels.

In ranging-based methods, a UWB positioning system has high accuracy and low latency due to its high frequency, moreover it is a low power consumption system and keeps stable positioning over the long-term. Therefore the UWB system is popular in indoor localization research, and numerous localization algorithms or synchronization algorithms have been proposed to achieve higher accuracy. Unfortunately, nothing is perfect; UWB localization is a high-cost system that needs at least three expensive anchors to realize 2-dimension (2D) positioning, and the UWB signal suffers from Non-Line of Sight (NLoS) which deteriorates the positioning results and increases the positioning cost concurrently (there is a requirement to add more anchors to alleviate NLoS). Many researchers use NLoS signals to implement localization based on prior knowledge of LOS/NLOS. However, in real application, it is hard to acquire prior knowledge and use NLoS knowledge to enhance localization results. Sobron et al. [16] adopted two correlation-based approaches to estimate time of arrival (TOA) under the assumption of the identification of an NLoS/Los channel. Yang [17] proposed a Sparse Pseudo-input Gaussian Process (SPGP) based on the NLoS method, which has low complexity compared with conventional approaches. Chen et al. [18] investigated NLoS mitigation under different machine learning methods such as long short-term memory (LSTM), convolutional neural network (CNN), and deep neural network (DNN). In some fusion strategies, the data under an NLoS scenario are discarded directly. Tian et al. [19] designed an Inertial Navigation System (INS) and UWB fusion system; in the calculation of a UWB base location stage, the UWB signals under NLoS scenario were discarded to achieve more reliable results. Joung et al. [20] used a CNN to estimate Time of Arrival (TOA) only from monochrome pictures constructed with received signals, which shows that a CNN has good potential for use in a positioning system. In [21], The Support Vector Machine (SVM), using channel impulse responses, was utilized to identify the NLoS of a UWB positioning system, and the experiment showed that it had a high classification accuracy. Yu et al. [22] proposed an equality constrained Taylor series robust least squares (ECTSRLS) technique to suppress NLoS ranging errors.

In order to implement high accuracy indoor positioning, alleviate the update frequency of the fingerprint database, and fully exploit the advantages of fingerprint-based methods and ranging-based methods, we proposed a CNN-based indoor localization system whereby the mapping relationship between the characteristics of received signals and location is stored in the weight parameters of the CNN, which could save storage cost, to some extent. Moreover, the UWB ranging is adopted to track change in the dynamical environment by refreshing weights of the CNN through errors backpropagation. To alleviate NLoS interference, the Support Vector Regression (SVR) and IMU data are designed to achieve a UWB ranging recovery. The main contributions of this paper are listed as follows:

- The proposed system is a fully automatic scheme, which means that the system can be used to localize position directly, and can work without any former preparation, e.g., manual measurement of the UWB anchor position and fingerprint location, etc. It only costs a short amount of time to initialize the whole system.
- To the best of our knowledge, this system is the first to use IMU measurements to correct UWB ranging. It highly expands the use of a UWB signal in an NLoS scenario and solves the localization problem in a harsh environment using ranging measurements.
- The proposed system can adapt to a dynamical environment properly, relying on the corrected UWB ranging feedback, and can provide a comparatively stable localization result over the long term.
- Using corrected UWB ranging measures can reduce the amount of CNN training (fingerprint database updating). Moreover, the position of the used UWB anchor can be ignored in this system (only ranging measurements are needed).

The remainder of this paper is organized as follows. In Section 2, the related work is introduced from the aspect of machine learning and UWB fusion system, and the detailed description of our proposed system is shown in Section 3. The corresponding experiment designs, as well as analysis of experiment results, are provided in Section 4. In Section 5, we discuss our system according to experimental results, and the conclusion is given in Section 6.

## 2. Related Work

### 2.1. Machine-Based Localization

The use of machine learning methods for indoor localization has attracted considerable attention in recent years. Dou et al. [23] formulated the indoor localization problem as a Markov Decision Process (MDP), using Deep Q Learning (DQL) to bisect the whole positioning space in 3-Dimension (3D); this method had low complexity and flexible localization resolution. Song et al. [24] used K-Nearest Neighbor (KNN) to estimate location after comparing the Time-reversal Resonating Strength (TRRS) and Euclidean distance between reference fingerprints and target fingerprints. Carrera Villacrés et al. [25] designed a particle filter based reinforcement learning localization system fusing Wi-Fi fingerprint and IMU-based PDR; this system can change its positioning model according to channel types (LoS/NLoS), and has high localization accuracy and strong robustness compared with traditional methods when the propagation model matches the real environment well. Chen et al. [26] used two CNN to realize indoor localization, called a two-stage CNN deep learning approach; one was used to identify the inherent features of an environment, based on first CNN recognition results (choosing an appropriate positioning model), the other one was applied to realize localization. Zhou et al. [27] proposed a method named AdapLoc which was based on one-dimensional Convolutional Neural Network (1D-CNN) to dynamically adapt to environmental change, and the evaluation experiment verified the effectiveness of AdapLoc. Zhao et al. [28] designed a hybrid convolutional autoencoder neural network to extract the features of location-related signals, and the experiments showed that the convolutional autoencoder neural network not only worked well in a real world dataset but also had anti-noise ability and low latency (average 4 ms). Chen et al. [29] proposed a Dilated CNN prediction and SVR correction Wi-Fi localization method, which had good real-time performance with only one RSS collection at each position. Consequently, it needed a large number of Accessible Points (64 for 8 × 8 size picture) to realize positioning with high accuracy.

In machine learning, deep learning is the most popular research field; one of the excellent characteristics of deep learning is the ability to inherently extract deep representative features in given data samples. In deep learning, CNN is the most popular localization tool among various machine learning methods; CNN has the ability to learn spatial features from data samples, thus, the temporal series signals can be changed into spatial series signals which can be processed by CNN. However, these deep learning methods have a latent assumption that the distribution of signal keeps stable in the long-term range. In order to follow the change of environment, the single UWB anchor-based ranging system is used in our proposed system.

### 2.2. UWB Fusion Localization

As formerly stated, a UWB localization system is a high-accuracy but expensive positioning system. Unfortunately, to alleviate the interference of NLoS in practical applications, the number of UWB anchors is much more than the theoretical number in real applications. Therefore, some researchers are devoted to a hybrid system based on a single UWB ranging with only one anchor. Tian et al. [18] utilized a Particle Filter (PF) to fuse PDR and UWB ranging; moreover, the anchor position was estimated in the initial stage to show that sensor drift was not significant. A ranging error model was then modified to implement PDR and UWB fusion using PF [30] to mitigate interference of NLoS. Cao et al. [31] designed a UWB ranging and IMU fusion algorithm which used UWB ranging and heading (provided by IMU) to calculate target speed, and an extended Kalman filter to fuse IMU and UWB ranging constricted by estimated speed. Li et al. [32] used an extended Kalman filter to fuse a UWB localization system (not ranging) and IMU, and they also discussed the fusion system under LoS and NLoS environments. Shi et al. [33] used commercial IMU and UWB ranging to calculate anchor coordinates which simplified the deployment of the UWB system, after which the UWB measurements and inertial measurements were fused by a tightly-coupled error-state Kalman filter. Xu et al. [34] proposed a fix-lag extended finite impulse response smoother (FEFIRS) to implement UWB and INS data tight fusion, and the results showed that FEFIRS had higher robustness and accuracy compared with traditional Kalman-based schemes.

These UWB fusion systems mostly rely on pre-knowledge of the anchor position and an NLoS environment transition model. The measurement of a UWB anchor position is a time consuming process, which will limit the use of UWB localization systems on a large scale. Moreover, though the NLoS model could provide stable positioning results, it cannot adapt to the dynamical environment.

## 3. Proposed Fusion System

This section gives an overview of the proposed positioning system followed by the description of some key components in detail. In Wi-Fi localization technology, compared with the variance of Received Signal Strength Indicator (RSSI), the CSI information has more stable properties and finer grained accuracy [35]. Therefore, the CSI is used to realize indoor localization in this paper.

### 3.1. Overview

**Figure 1:**
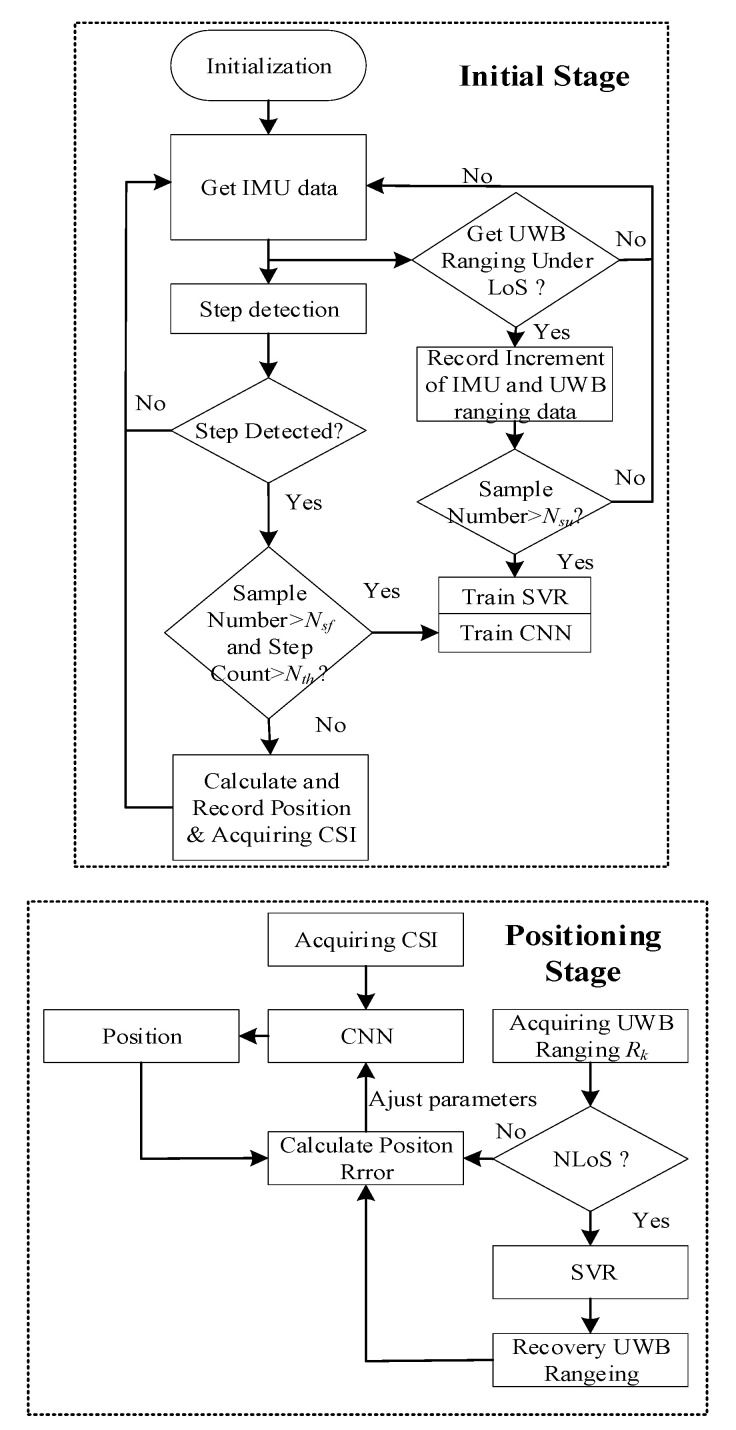
The structure of the proposed system. *N_sf_*, *N_su_* is the sample number of CNN and SVR, respectively. *N_th_* is the threshold of the step number. *R_k_* is the *k*-th UWB ranging.

The whole system is divided into two stages: an initial stage and a positioning stage, as shown in Figure 1. In the initial stage, a tester equipped with an IMU sensor and a UWB tag (mounted tightly) starts from a fixed point (a known coordinate such as an entrance), and the initial stage consists of two parts: CNN training and SVR training. During CNN training, if the step is detected in the Step detection block using the IMU data, the system will calculate the current location and record position-related fingerprints when the sample number and step count are less than their threshold *N_sf_* and *N_th_*. It should be emphasized that the sample number *N_sf_* is much larger than *N_th_*, thus, if the step count reaches the threshold *N_th_* while the sample number is insufficient, it should return to a fixed point to restart the PDR position calculation and fingerprint collection along a different test line until the sample number is satisfied. This manipulation not only keeps the high accuracy of PDR localization but also ensures quantity and quality of the training sample for the CNN. During SVR training, the data flow is triggered by new IMU data. The system will record the increments of IMU and UWB ranging data in pairs for SVR training when the UWB data are collected under LoS environment; after the sample number reaches its threshold *N_su_*, the SVR begins to train.

In the positioning stage, the system uses a current fingerprint to estimate the corresponding location; at the same time, the recovered UWB ranging under NLoS or raw UWB ranging under LoS is utilized to calculate CNN positioning error according to CNN estimated position, and then the parameters of CNN are adjusted using estimated errors. We will elaborate on the key technologies and components in subsequent sections.

### 3.2. CNN Training

In this part, the basic technologies utilized in training sample collecting are step detected and position calculated using IMU data, which have been researched sufficiently. The specific values of the sampling number and step count threshold *N_sf_*, *N_th_* will be discussed in the experiment preparation section.

#### 3.2.1. Step Detection

Step detection can be treated as Stance detection, because feet touch the ground alternately, which generates a tiny zero-velocity interval per step. There have been many step detection algorithms developed in recent years, but most have been improved based on [36], with more complex and strict constraints. For example, Liu et al. [37] proposed an example of a robust step detection algorithm that reduced the false-detection and over-detection of steps well. For simplicity, the step detection algorithm in [36] is utilized in our system, and a simple description of its three constraint conditions (*C*1, *C*2, *C*3) are given as follows:

(1) $$C1=\left\{ \begin{array}{l} \begin{array}{cc} 1 & th_{a\min}<\left|a_{k}\right|<th_{a\max} \end{array}\\ \begin{array}{cc} 0 & otherwise \end{array} \end{array}\right.,$$

(2) $$C2=\left\{ \begin{array}{l} \begin{array}{cc} 1 & \sigma_{a}>th_{\sigma_{a}} \end{array}\\ \begin{array}{cc} 0 & otherwise \end{array} \end{array}\right.,$$

(3) $$C3=\left\{ \begin{array}{l} \begin{array}{cc} 1 & \left|\omega_{k}\right|<th_{\omega } \end{array}\\ \begin{array}{cc} 0 & otherwise \end{array} \end{array}\right..$$

The thresholds $th_{a\min}$ and $th_{a\max}$ are lower bound and upper bound respectively; $\left|a_{k}\right|$ denotes the square root of *k*-th acceleration sampling; $\sigma_{a}$ denotes the root mean-variance of acceleration under a given window scale; $th_{\sigma_{a}}$ is the selected threshold; $\left|\omega_{k}\right|$ represents the square root of *k*-th gyroscopic sampling; and $th_{\omega }$ is the corresponding threshold.

#### 3.2.2. Position Calculation

The PDR is an efficient localization algorithm, it can iteratively calculate target position using IMU heading data and step length:

(4) $$\left[\begin{array}{c} x_{k}\\ y_{k} \end{array}\right]=\left[\begin{array}{c} x_{k-1}\\ y_{k-1} \end{array}\right]+\left[\begin{array}{c} \cos\theta \\ \sin\theta \end{array}\right]\times L_{s},$$

(5) $$L_{s}=k_{s}\times h\times \sqrt{f_{s}}.$$

$x_{k}$ and $y_{k}$ are the coordinate values of *k*-th step in a planar coordinate system. $L_{s}$ denotes the step length in which $k_{s}$, h, and $\sqrt{f_{s}}$ are scaling factor, pedestrian height, and corresponding walking frequency respectively.

In common scenarios, it is hard to achieve high accuracy positioning results between different testers. However, our proposed system can guarantee that only one person is needed to initialize all systems in different scenarios at the system deployment stage and the positioning stage. The proposed system abandons the PDR algorithm, avoiding tedious PDR correcting processes between different people. In summary, the fixed person initializes the whole system, after which the system can be available for everyone. This is the superiority of our system compared with PDR.

#### 3.2.3. CNN Regression Model

**Figure 2:**
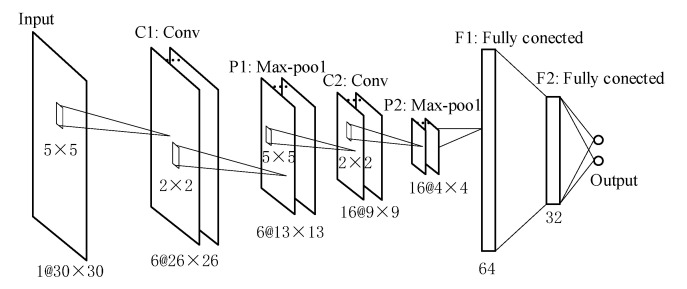
The structure of CNN used in this paper.

For simplicity without loss of generality, a traditional CNN consists of two convolution-pooling layers (shown in Figure 2), to realize position calculation and verify the effectiveness of the proposed system. The method is simplified in references [38,39]. This CNN consists of two 5×5 convolutional layers (C1 and C2), two max-pooling layers (P1 and P2) with stride 2, and three fully connected layers (F1, F2, F3). The output layer (F3) has two output nodes and each node outputs a corresponding coordinate value.

As is depicted in Figure 1 flow chart, the feedback error signals are different between the initial and positioning stages. In the initial stage, the feedback signals are calculated with positions of reference points in database and CNN estimated positions, thus the loss function Φ is defined as follows:

(6) $$\Phi =\frac{1}{N}\sum_{i=1}^{N}{\left\|y_{i}-f(x_{i};w)\right\|}_{2}^{2},$$

where N denotes the total number of CNN training samples, $y_{i}$ is the corresponding label of *i*-th training sample $x_{i}$, and $f(x_{i};w)$ is the output of the CNN regression model under the weight vector of w. The Stochastic Gradient Descent (SGD) is chosen to train weight w, and the weight update rule is:

(7) $$v_{i+1}:=m\cdot v_{i}-d\cdot \kappa_{i}\cdot w_{i}-\kappa_{i}{\langle \frac{\partial \Phi }{\partial w}|\begin{array}{c} {}_{w_{i}} \end{array}\rangle }_{D_{i}},$$

(8) $$w_{i+1}:=w_{i}+v_{i},$$

where $v_{i}$ is the *i*-th momentum variable, and m and d denote the constant momentum coefficient and weight decay respectively. The $\kappa_{i}$ is the learning rate decaying with a nonlinear rate. ${\langle \partial \Phi /\partial w|w_{i}\rangle }_{D_{i}}$ denotes the *i*-th iteration of loss function derivation with respect to w, on batch $D_{i}$, evaluated at $w_{i}$.

In the positioning stage, the CNN provides the predicted localization result and its weights are adjusted according to UWB ranging. The adjustment process can be regarded as environment characteristic tracking because the weights of CNN represent environment characteristics. Thus, the CNN-based system can theoretically realize long-term positioning through environment characteristics tracking using UWB ranging. In this stage, the corresponding loss function defined in positioning stage is:

(9) $$\Phi_{p}=\frac{1}{N_{p}}\sum_{i=1}^{N_{p}}\left|{\left\|ap-f(rx_{i};w)\right\|}_{2}^{2}-r_{UWB}^{2}\right|,$$

where $N_{p}$ is the number of localization targets, $f(rx_{i};w)$ denotes the output of CNN with real-time fingerprint measures $rx_{i}$ and parameter w. $r_{UWB}$ is the corrected UWB ranging\raw UWB ranging (under NLoS\LoS), with the SGD equally used in this stage. ap is the coordinate of UWB anchor which can be estimated through UWB ranging in initial stage [19].

The scheme of ap position estimation is described briefly as follows: firstly, the pedestrian locations are calculated using PDR while recording corresponding UWB ranging measures in the initial stage. After that, an empirical power metric of UWB signal [40] is utilized to sort the UWB power measures. Following that, three non-collinear PDR positions with the best UWB ranging quality are chosen. Finally, the anchor position is solved by trilateration using three selected PDR positions and UWB ranging pairs. The empirical UWB ranging power metric in unit of dBm is defined as:

(10) $$P_{diff}=P_{RX}-P_{FP}=10\times \log_{10}(C\times 2^{17}/F_{1}^{2}+F_{2}^{2}+F_{3}^{2}),$$

where $P_{RX}$, $P_{FP}$, C, $F_{1-3}$ are the total received power, First Path (FP) power and the amplitude of three points defined in [19], respectively. In this work, the UWB system equipped with DW1000 chip is used to measure distance. Thus, the values of C and $F_{1-3}$ can be achieved in the registers of DW1000 chip. Reference [40] suggests that the channel is likely to be under LOS state when $P_{diff}$ is greater than 10 dBm, while the channel is LOS when $P_{diff}$ is less than 6 dBm.

### 3.3. SVR Training

Although the pedestrian trace can be calculated through strapdown IMU assisted with UWB ranging correcting, it is a time-consuming and error-accumulating process; thus, it is hard to estimate high accuracy and long term trace, especially using commercial IMU. In the proposed system, the IMU is used in an indoor environment; compared with large-scale applications such as car or plane tracking, it is a tiny-scale application. Thus, the navigation frame can be treated as a fixed frame; moreover, low-cost IMU cannot sense some physical effects, such as the Earth’s curvature, rotation, etc., [41]. The UWB signals also suffer from object blockage in NLoS scenarios, while IMU is not affected by NLoS; moreover, NLoS and LoS always appear alternately. Therefore, the IMU data can be used to correct raw UWB ranging under LoS scenario, and can be verified through IMU trace formulas and distance formulas. The simplified relationship of position calculation between IMU and UWB ranging is:

(11) $$\begin{array}{cc} {\left\|P_{ANC}^{n}-P_{IMU(m)}^{n}\right\|}_{2} & =R_{UWB(m-1)}+\Delta R_{UWB(m)}\\ & ={\left\|P_{ANC}^{n}-P_{IMU(m-1)}^{n}\right\|}_{2}+\Delta R_{UWB(m)} \end{array},$$

where the $\Delta R_{UWB(m)}$ is the UWB ranging increment compared with the last ranging measurement, it relates with IMU position $P_{IMU(m)}^{n}$ and $P_{IMU(m-1)}^{n}$; the $P_{IMU(m-1)}^{n}$ is the position of IMU in last updating cycle, which is already known. The $P_{IMU(m)}^{n}$ is:

(12) $$P_{IMU(m)}^{n}=P_{IMU(m-1)}^{n}+\Delta P_{IMU(m)}^{n},$$

in which the $\Delta P_{IMU(m)}^{n}$ is:

(13) $$\Delta P_{IMU(m)}^{n}=\frac{v_{m-1}^{n}+v_{m}^{n}}{2}T_{s}.$$

Due to the limits of low-cost IMU, the $v_{m}^{n}$ can be:

(14) $$v_{m}^{n}=v_{m-1}^{n}+\Delta v_{sf(m)}^{n}+g^{n}T_{s},$$

where,

(15) $$\Delta v_{sf(m)}^{n}=C_{b(m-1)}^{n}(\Delta v_{m}+\frac{1}{2}\Delta \theta_{m}\times \Delta v_{m}),$$

(16) $$C_{b(m-1)}^{n}=C_{b(m-2)}^{n}C_{b(m-1)}^{b(m-2)},$$

(17) $$C_{b(m-1)}^{b(m-2)}=I+\frac{\sin\phi_{ib(m-1)}^{b}}{\phi_{ib(m-1)}^{b}}(\phi_{ib(m-1)}^{b}\times )+\frac{1-\cos\phi_{ib(m-1)}^{b}}{\phi_{ib(m-1)}^{b}{}^{2}}{\left(\phi_{ib(m-1)}^{b}\times \right)}^{2},$$

(18) $$\phi_{ib(m-1)}^{b}=(\Delta \theta_{(m-1)1}+\Delta \theta_{(m-1)2})+\frac{2}{3}\Delta \theta_{(m-1)1}\times \Delta \theta_{(m-1)2}.$$

where the subscript m denotes *m*-th updates of corresponding variables, the letters *i*, *n* and *b* are the abbreviation of navigation frame, body frame and inertial coordinate frame respectively, with $T_{s}$ the update interval. $P_{ANC}$, $P_{IMU}$, $\Delta P_{IMU}$, $R_{UWB}$, $\Delta R_{UWB}$ are respectively the constant coordinate of UWB anchor, the position of IMU, the position increment of IMU, the UWB ranging measure and the increment of UWB ranging measure. $\Delta v_{sf}^{n}$ is the specific force increment in *n*-frame and Δv is the increment of specific force sampling from the accelerometer in a sampling period. $\theta_{(m)1-2}$ denotes twice sampling results in *m*-th updating period. Ignoring intermediate variables in Equations (12)–(14), it can be seen that the increment of UWB ranging measure relates to velocity increment Δv and angular increment Δθ. Therefore, *m*-th UWB ranging can be expressed with the initial location (last position under LoS) and corresponding increments Δv and Δθ.

From the formulas listed above, the relationship between UWB ranging increment and IMU reading increment is high dimensional, and the analytic solution is hard to achieve. Therefore, in order to realize UWB ranging correction and alleviate the error accumulation effect, the powerful ability of inherent characteristic extraction of SVR is applied to estimate the increment of UWB ranging under NLoS environment. Due to highly non-linear characteristics of radial basis function (RBF), the RBF is chosen as kernel function in SVR. Equation (11) reveals that the increment of the UWB range depends on the location of the anchor and the sensor. However, inspired by Equations (12)–(18), the IMU reading increments (gyroscope and accelerator) and last position of the sensor (where the UWB mobile node and IMU are fixed tightly) are chosen as the input data of the SVR to predict the corresponding UWB ranging increments in every sampling period, because the positions of the anchor are fixed parameters and their location information implicitly exists in data tuples of the training database, i.e., increment of IMU reading (input), last position of sensor (input), and UWB ranging increment (target). In other words, the position of the UWB anchor and other fixed parameters are reflected in the weights of SVR after undergoing offline training.

**Figure 3:**
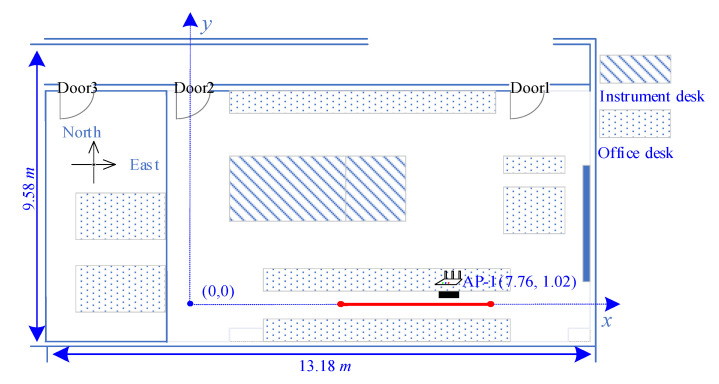
The layout of the experiment area.

**Figure 4:**
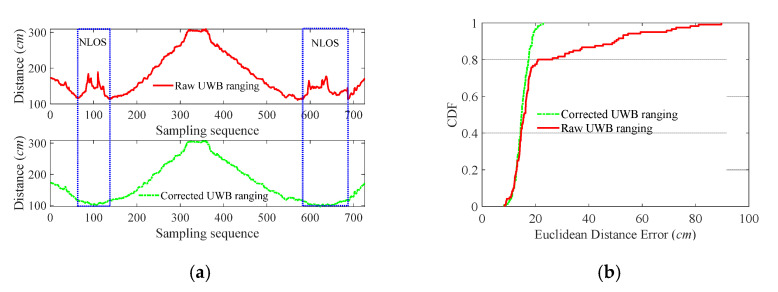
The correction of UWB ranging measurements under NLoS (about 20%), the raw UWB ranging marked with a red line and the corrected UWB ranging marked with a green dash-dotted line. (**a**) the UWB ranging tests. (**b**) the Cumulative Distribution Function (CDF) of Euclidean distance errors.

If the UWB range is measured under NLoS, learning from the former empirical power metric [40], the increment of the IMU reading and last sensor position are sent to SVR to predict the UWB ranging increment. As shown in the lower right part of Figure 3, there is a straight route (red line) and a metal baffle (black rectangle) between the red line and Access Point 1 (AP-1). Reciprocating along the red line, the corresponding raw UWB ranging measurements are shown in Figure 4 with red line, showing that the NLoS arouses fluctuation of UWB ranging. The SVR-corrected results are displayed with a green dash-dotted line which is consistent with the actual situation.

Moreover, we conducted a series of experiments to verify the validity of correction UWB ranging using SVR and IMU through changing the ratio of NLoS environment (adding baffles). The total moving distance was 7.2 m with 25 ranging points deployed evenly on the red trajectory, as shown in Figure 3. The results of the experiment are listed in Table 1, showing that the SVR and IMU could reduce the mean ranging error by up to 30.40% even under a harsh environment (high NLoS ratio 80%). Though the (Standard Deviation) Std of correction ranging decreases with the increase in NLoS ratio, the correction scheme can still keep low mean and Std of error compared with raw UWB ranging.

## 4. Positioning Experiment and Analysis

### 4.1. Preparation

Figure 3 shows the layout of the experiment environment, which is a 13.18 m × 9.58 m hybrid office room with some instruments and office supplies. All experiment data were processed on a computer with Intel Core i5-10400F CPU, 16 GB RAM and NVIDIA GeForce RTX 2060.

#### 4.1.1. PDR Parameters Setting

**Figure 5:**
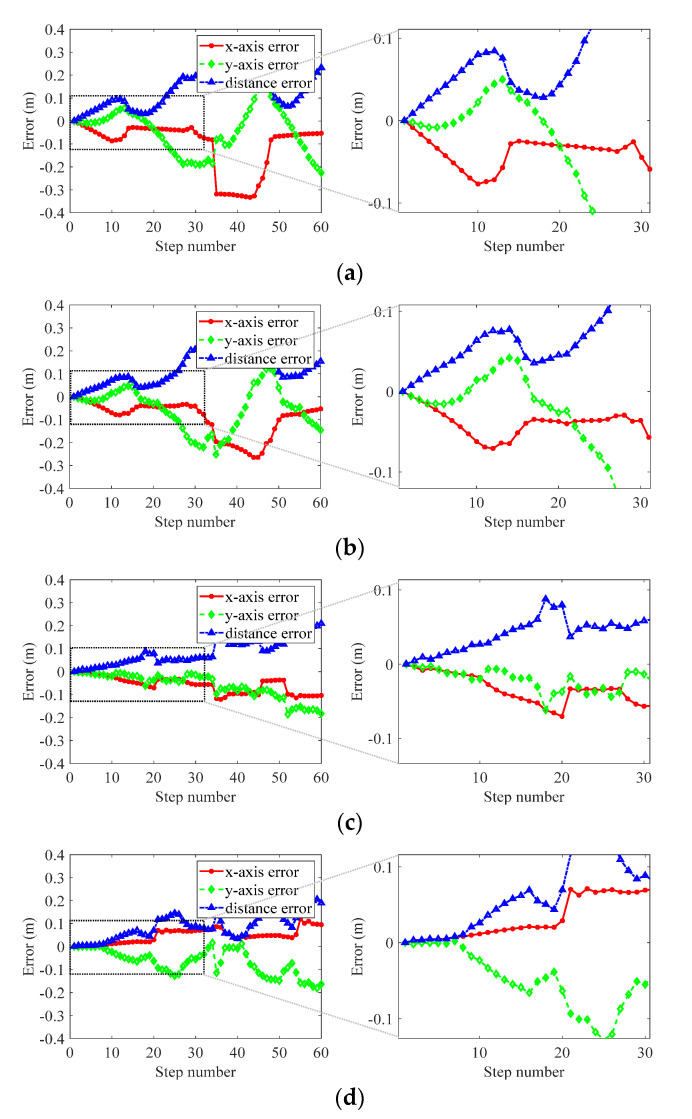
PDR tracking accuracy changes with step number. The figures (**a**–**d**) are four different testers’ PDR tracking accuracy.

Four different people (three men and one woman) mounted with an IMU, walked along a fixed rectangular route (34 steps per circle) to confirm the value of step threshold Nth using a basic PDR algorithm; the PDR tracking results shown in Figure 5 illustrate that the PDR algorithm had high localization accuracy (below 0.1 m) within 20 steps. Therefore, the value of step threshold Nth is set to 20 in the proposed system. For simplicity and generality, basic PDR and low-cost IMU sensors are utilized in this system. However, a high step threshold can be achieved using a more expensive sensor or a better PDR algorithm. The parameters of IMU utilized in this system are listed in Table 2.

#### 4.1.2. CNN Parameters Setting

**Figure 6:**
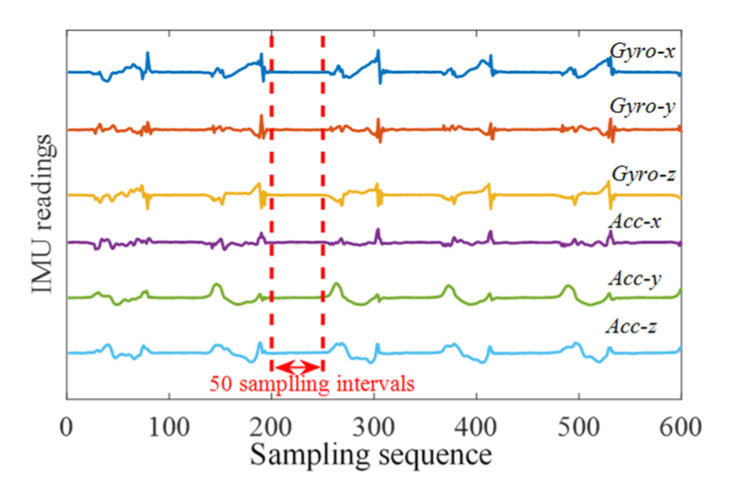
The tester’s ground contact time of walking.

The input size of the CNN relates to the number of APs and the length of sampling sequences. In this paper, only one AP was used to collect a CSI fingerprint in the system. In order to select an appropriate sampling number and keep correlation in sampling sequence (sequential signals are received in a fixed point while the pedestrian is walking), the signal transmission interval and the ground contact time (interval between heel-strike and heel-off) should be considered. As depicted in Figure 6, the tester’s ground contact time of walking (walking speed was about 1.8 m/s, which can satisfy most motion modes in an indoor scenario) had at least 50 sampling periods (500 ms) when the sampling period of the IMU was 10 ms (100 Hz).

**Figure 7:**
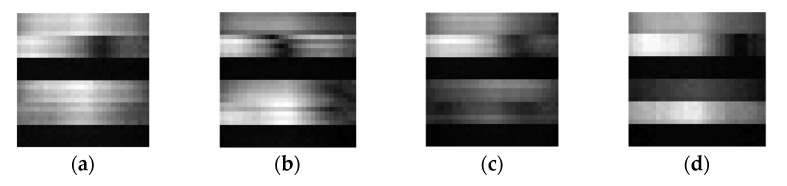
The CSI amplitude grayscale images. The figures (**a**–**d**) show that the CSI is unstable in the same place of different time points.

The CSI is described by a function, i.e.,

(19) $$H=\left|H\right|e^{j∠H},$$

where |H| and ∠H are the corresponding amplitude and phase, respectively. In this paper, the average interval between adjacent received CSI packet was about 100 ms, which means that at least 5 packets could be received during each foot contact with the ground. Moreover, it had 6 channels (2 transmitting antennas, 3 receiving antennas) in each data packet and each channel had 30 subcarriers. Therefore, a 30 × 30 (5 packets × 6 channels × 30 subcarriers) matrix of amplitudes could be formed to a location-related CSI image as shown in Figure 7, reflecting a weak signal in third receiving antennas which acted as auxiliary antennas. 

In the fingerprint collection stage, people walked along given routes and remained stationary at each step for around 10 s, while the fingerprint data was collected. We collected CSI at 40 locations with 100 × 40 size of training and five received packets were randomly picked to test performance in the changed environment. The values of the experiment parameter are listed in Table 3.

### 4.2. Test Evaluation

In this part, all metal doors (Doors 1–3 shown in Figure 3) were kept in an open state when fingerprint data was collected. In the initial environment (denoted as En1), a tester randomly walked 280 steps in the experiment area (covering nearly all the area) to collect training samples. In order to improve the stability of the CNN system, the collected 280 samples were expanded to 4000 samples by adding White Gaussian Noise (WGN) which could improve robustness of CNN feature extraction [42]. After that, we changed the environment by adding an NLoS condition, i.e., object blocking and pedestrian stochastic walking (En2) and further closing all doors (denoted as En3). Then, another tester walked randomly in the three environments to evaluate the localization system.

#### 4.2.1. Basic Performance Test

**Figure 8:**
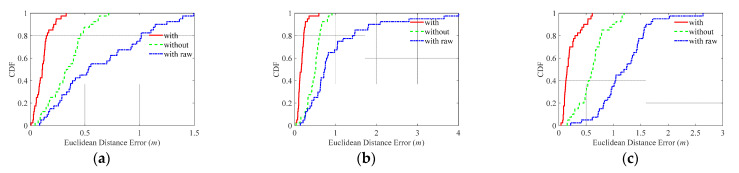
Localization results in different scenarios. (**a**) Localization results in En1. (**b**) Localization results in En2. (**c**) Localization results in En3.

**Figure 9:**
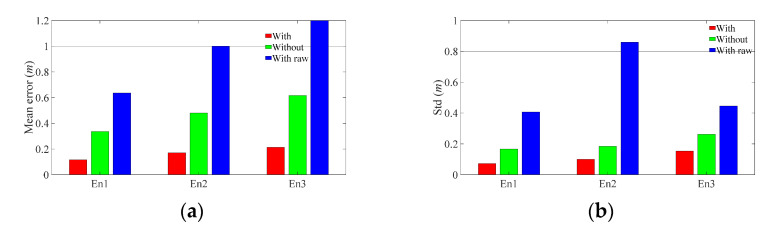
Statistics of location errors. (**a**) Mean error. (**b**) Standard deviation of error.

The Euclidean distance between predicted location and true location was used as localization error (m). The corresponding cumulative distribution function (CDF) of the localization errors and comparisons was computed and is given in Figure 8 and Table 4, in which the character(s) ‘With’, ‘Without’, and ‘With raw’ respectively denote calibrated CNN localization result with corrected UWB ranging, without UWB ranging, and with raw UWB ranging. It reflects that the calibrated UWB ranging can considerably improve the CNN-predicted results compared with ‘Without’ and ‘With raw’ schemes. Moreover, the localization performance on mean errors and Standard deviation (Std) are given in Figure 9 and Table 5. Figure 9 and Table 5 indicate that ‘With’ scheme has higher and more stable localization results compared with other schemes in terms of mean error and corresponding Std. It also reveals that the accuracy of UWB ranging is the key to performance improvement. More specifically, raw UWB ranging without correction may incur performance degradation compared with no UWB assisted scheme (‘Without’ scheme), and the high-accuracy UWB measurement can improve the localization performance.

The quantitative comparisons between ‘With’ and ‘Without’ schemes in Table 5 show that the error reduction percentile of ‘With’ scheme on ‘Without’ scheme stabilizes at around 65%, and the corresponding Std reduction is more than 40% in the three different scenarios.

#### 4.2.2. Daily Localization Performance Test

**Figure 10:**
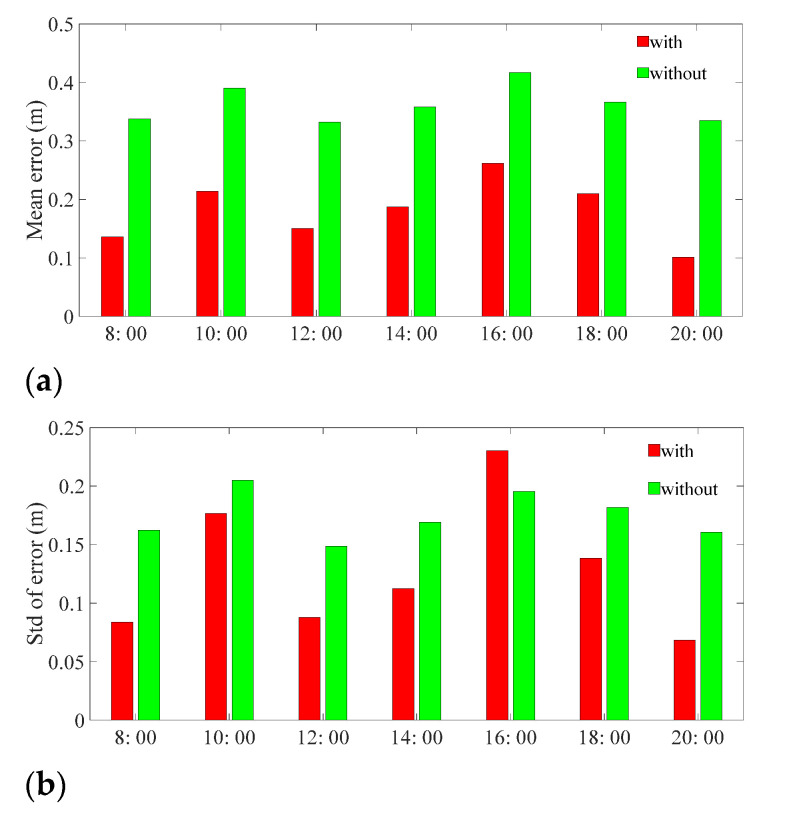
Positioning performance in the daily environment. (**a**) Mean error of localization. (**b**) Std of localization error.

In order to evaluate the performance of the localization system in daily time, 2000 (steps) location-related fingerprint data were collected from three testers (walking along preset test points) every 2 h from 8:00 A.M. to 8:00 P.M. During the data collecting stage, there were other students also working or walking in the testing environment. The mean and Std of localization error are shown in Figure 10.

As depicted in Figure 10, the mean localization error of the proposed scheme (‘With’) is lower than that of the CNN directly-predicted results (‘without’) in every time interval, which verifies that the proposed system can reduce the localization error effectively. Moreover, the positioning error of the proposed scheme is lower than 0.25 m and the error of the corresponding CNN prediction scheme (‘without’) is bigger than 0.35 m in all test intervals. Due to people body interference and changes in the tiny environment, the localization error varies with time; it is obvious that the localization system has the maximum positioning error at 16:00 (people are most tightly concentrated in this period of time) and the minimum positioning error at 20:00 (when there is minimal human interference).

#### 4.2.3. Long-Term Localization Performance Test

**Figure 11:**
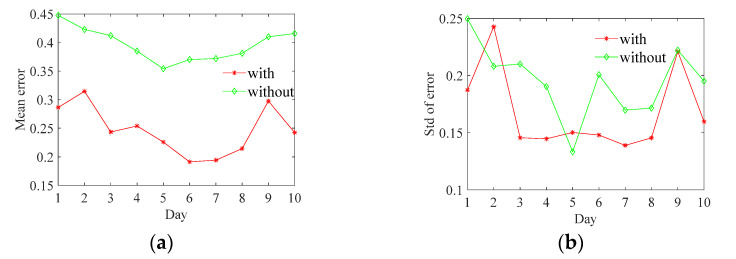
The long-term localization errors. (**a**) Mean error. (**b**) Std of error.

To study the long-term localization performance of the proposed system, the fingerprint information was collected over 10 consecutive days at 12:00 and 16:00 every day. The size of test data comprised 240 locations (240 steps each, for three people, along fixed points), and the means and Std of localization errors are displayed in Figure 11.

From Figure 11, we can conclude that the proposed UWB ranging calibrated system (‘with’) has high positioning accuracy (lower than 0.32 m) in long-term and stable performance (Std within 0.25 m). As shown in Figure 11b, due to occasionally wrongly predicted UWB ranging and a system sensitive to wrong UWB ranging (refer to ‘with raw’ scheme shown in Figure 9), the Std of the proposed system has a larger error Std than that of the CNN-direct prediction at day 2 and day 5. However, the proposed system can recalibrate the positioning results and keep them stable using subsequent high-accuracy UWB ranging measures, which is satisfied with practical applications.

#### 4.2.4. Noise Injection Test

To evaluate the robustness of the proposed system, we injected the Gaussian noise (0, σ2) into the fingerprint gray image, where the Mean Square Error (MSE) σ is the deviation of image gray ranging from 0 to 52.

**Figure 12:**
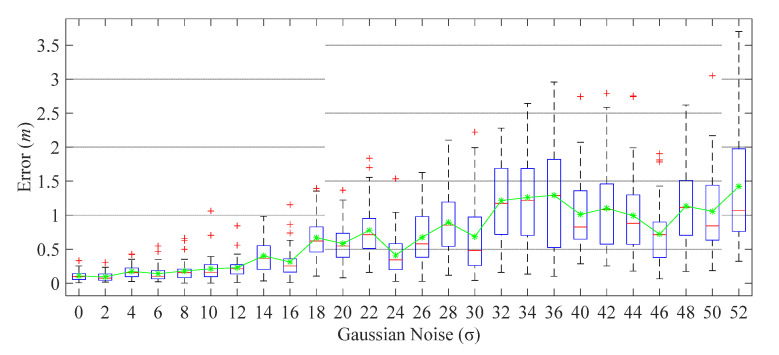
The positioning error under Gaussian noise injection. The green curve is the mean error.

The experimental results depicted in Figure 12 show that the localization system can still keep most of the mean localization error (75%) under 0.5 m, and 1 m when σ ≤ 12, σ ≤ 26. Importantly, the whole localization accuracy is considerably stable (the biggest outliers are no more than 0.7 m) when σ ≤ 8. This experiment shows that the proposed system has a certain degree of anti-noise attack ability.

#### 4.2.5. Impact of Parameters on the Positioning Stage

**Figure 13:**
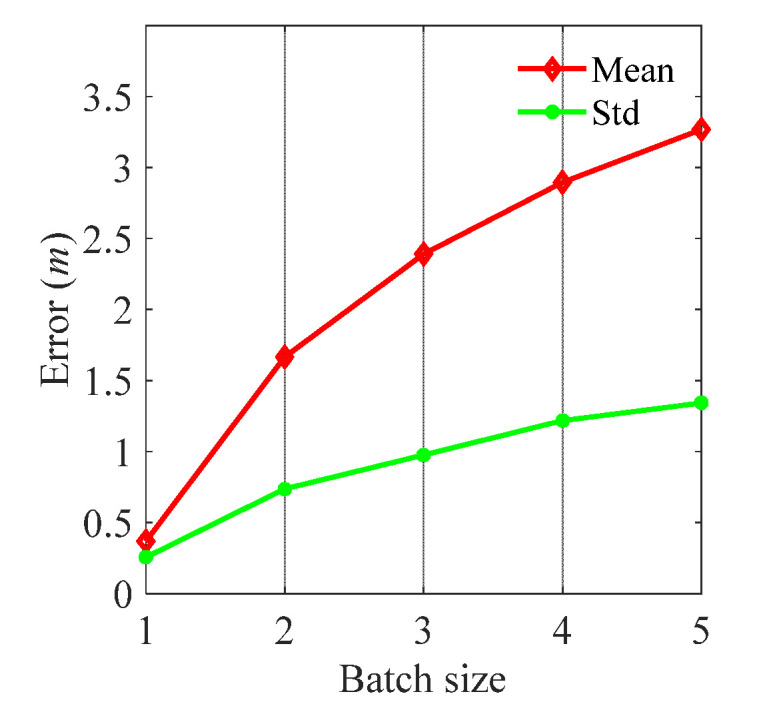
The positioning errors vary with batch size.

In the initial stage, the training of the CNN is common training progress, which is stable and predictable. However, compared with the initial stage, the positioning stage has minor adjustment progress and is vulnerable to basic parameters. In this part, the important parameters of the CNN training in the positioning stage are discussed including batch size and number of test points. Localization errors are calculated under different batch sizes while keeping other parameters consistent. The experimental results are shown in Figure 13.

Figure 13 shows that localization errors increase with batch size, and the stability of positioning decreases with batch size (Std increases with batch size). Since the CNN has been fully trained in the initial stage, the mapping parameters of CNN can represent initial environment characteristics. Though the environment has changed in the positioning stage, the mapping parameters of the CNN only need minor adjustment. However, the larger the batch size, the more mapping parameters modification required, which could increase the error of other positioning points. Therefore, the batch size should be set as 1.

**Figure 14:**
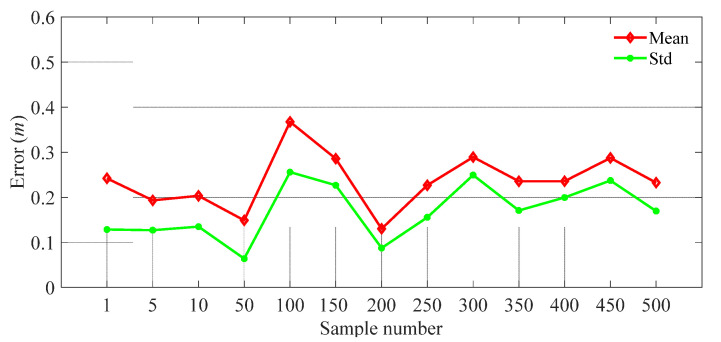
The impacts of sample number.

To evaluate the impacts of the sample number on the positioning error, we calculated the localization error using different test numbers as shown in Figure 14. This indicated that the localization error was insensitive to the test number, i.e., the positioning accuracy remained relatively stable (within 0.4 m) when the number of samples increased. Moreover, as shown in Table 6, the update time (i.e., time of giving calibrated results) increased with the sample number. Therefore, the proposed system can realize online learning\calibrating (in the positioning stage) with high position accuracy, and also has the potential to locate multiple people where the sample size is equal to the number of people.

#### 4.2.6. Comparison with Recent Related Works

The proposed localization system is compared with the latest related research listed in Table 7. It is obvious that these recent results considerably contribute to an indoor positioning system. Based on RSS or CSI information, most of these works adopt a classification strategy to locate target; multiple information fusion and trilateration calculation are also adopted as positioning strategies. Unlike cited relevant works, the CNN regression prediction is used in our system to provide continuous target coordinates using its mapping power.

Though these works achieve good localization results based on respective positioning conditions, the long-term positioning performance has not been tested or discussed except in [43]; the practical application of the positioning system mostly depends on the long-term localization ability, which is key to cost-saving. Due to a more fine-tuned location-related information (CSI) and UWB ranging calibration, our proposed system keeps the long-term localization error within 0.4 m, comparing with meter-level accuracy in [44].

As for the number of AP, these positioning systems rely on multiple positioning base stations, some of which reach hundreds, e.g., [43]. However, [45] achieves low localization error (0.2 m) using CSI and RSSI hybrid information provided by only one AP, but as it does not consider long-term property of the localization system, its positioning performance will deteriorate over time (environment change). Reference [44] has the largest positioning area with lower accuracy (meter level) using less AP, but is suitable for indoor positioning scenarios (with meter-level accuracy).

Compared with related works, our contribution to the area is not the most superior, but we managed to balance the cost and positioning accuracy, i.e., only 2 base stations (one is Wi-Fi, the other is a UWB anchor) are utilized to provide location-related information and calibration signals; and based on this basic information, the CNN regression model is utilized to realize continuous positioning with high localization accuracy over a long-term range. Moreover, the proposed system was tested in a large-scale room which is suitable for most scenarios.

**Table 7:** Comparison with recent localization works.

Reference	Short-Term Accuracy (m)	Long-Term Accuracy (m)	AP Number	Localization Information	Area	Strategy
[43]	3.91	3.91–4.49 *	~258 APs	RSSI (ratio)	12.5 m × 10 m	classification
[45]	0.20, 0.38	-	1	CSI + RSSI	6 m × 9 m	classification
[44]	2.6	-	14	RSS + INS	40 m × 100 m	fusion
[46]	0.1–3.5 *	-	At least 3	RSSI + Loss model	8 m × 8 m	trilateration
[47]	0.23–2.10	-	144	RSSI + magnetor	27.6 m × 12.8 m	classification
Ours	0.21	0.19–0.32	2 (UWB + CSI)	CSI + UWB + INS	10.1 m × 8 m	regression

* the data are not given in the original text and are inferred from the corresponding descriptions or charts.

## 5. Discussion

Although the proposed system can realize stable and robust localization, there are still some problems to discuss.

1. The proposal can save the cost of labor and system deployment. However, due to the high drift and noise interference of commercial IMU, the fingerprint collector must return to the fixed coordinate-known point (the entrance or some other given points) when the number of steps reaches the precision threshold of the PDR algorithm. As a result, the area of fingerprint collection is limited to a circle with the fixed point (the entrance or some other given points) as center, and straight PDR distance (within threshold of step number) as radius. This limit can be solved by adopting a more expensive IMU rather than adding an anchor to implement location-related fingerprint collection in a large area, and the equipment of fingerprint collection can still be reused to collect fingerprint in other interesting places.
2. In this paper, the UWB ranging measure is the key to following environment change and calibrating localization results, thus, the whole system is also vulnerable to an NLoS environment. In our design, machine learning is utilized to recover UWB ranging measures under an NLoS environment. Although the machine learning method can give a reliable result, it has to be retrained when the positioning environment or the position of the UWB anchor has changed. There are two solutions to avoid NLoS interference: discarding UWB ranging under NLoS or constructing a UWB transmitting channel model. In terms of the discarding method, the UWB calibrated function cannot work in NLoS, which limits the system’s practical application and reduces the stability of the system (the system frequently changes between calibrated and uncalibrated state). As for the latter solution, there are some UWB signal transmitting models under different blocking objects and these models can recover UWB signal well. However, in practical application, it is hard to design a valid transmitting model suitable for various or multiple blocking interferences. Additionally, the threshold of NLoS judgment needs to be devised in a different environment rather than using experiential value.
3. Unlike most works, the CNN regression model is used to predict location based on a gray image of CSI amplitude fingerprint. The essence of CNN prediction is the mapping function between position and CSI (similar to a signal transmitting model), which is sensitive to environmental change. For this reason, the UWB ranging measure is utilized to dynamically adjust the CNN predictions and weight parameters in this paper. Although the CNN is vulnerable to environmental change, it has its own superiority, i.e., outputting continuous location, which is the inbuilt advantage of realizing high-accuracy localization compared with the classification method.
4. In addition to the discussed and tested parameters, there are a large number of factors affecting positioning results in practical application, e.g., the size of the fingerprint image and the choice of length of Wi-Fi sequences, etc. Based on these dynamical factors, our future work will concentrate on a more comprehensive but efficient localization system.

## 6. Conclusions

In this article, a UWB ranging calibrated localization system based on a CNN regression model has been developed to realize high-accuracy indoor positioning. Specifically, the proposed system can track dynamical environment characteristics using UWB ranging measure, which can mitigate the effects of environmental changes on localization results. Moreover, the PDR algorithm is employed to save the cost of fingerprint collection and anchor deployment in the off-line stage. A series of experiments have been carried out to testify the priority of this system: these experiments show that the system has strong robustness and adaptability; furthermore, it has excellent short-term and long-term positioning ability with high localization accuracy (lower than 0.35 m) and stability (lower than 0.25 m). Finally, the noise injection test reveals that the gray images of fingerprints have a certain degree of anti-noise attack ability. All the experiments testify that the proposed system is effective in indoor positioning.

There are still several directions to further improve this work including optimization of the CNN for positioning, establishing a high-efficiency fingerprint, designing more intelligent localization structure, etc. Our future work will focus on these research points.

## Figures and Tables

**Table 1:** The error comparison between raw and correction UWB ranging.

Ratio of NLoS	Item	Mean (m)	Std (m)
20% NLoS	Raw UWB ranging	0.32	0.22
	Correction UWB ranging	0.18	0.04
	Correction ratio	43.75%	81.81%
50% NLoS	Raw UWB ranging	0.41	0.19
	Correction UWB ranging	0.24	0.11
	Correction ratio	41.46%	42.11%
80% NLoS	Raw UWB ranging	0.54	0.21
	Correction UWB ranging	0.37	0.20
	Correction ratio	31.48%	4.76%

**Table 2:** Parameter values of IMU.

Parameter	Value
Size	39 mm × 39 mm × 8 mm
Accelerator	3-axis, ±16 g
Gyroscope	3-axis, ±2000 dps
Sampling frequency	100 Hz
Resolution	<0.05°

**Table 3:** Values of experiment parameter.

Parameter	Value/Setting
Number of samples in CNN *N_sf_*	4000
Number of samples in SVR *N_su_*	4000
Step threshold *N_th_*	20
The kernel function of SVR	Radial Basis Function

**Table 4:** Comparison results in detail.

	Calibration	Percentile			Euclidean Distance Error (m)			
		50%	70%	90%	<0.2	<0.3	<0.5	<1
En1	With	0.11	0.13	0.21	84.76%	97.51%	100%	100%
	Without	0.33	0.43	0.54	25.42%	39.83%	87.58%	100%
	With raw	0.52	0.92	1.14	15.81%	27.90%	45.12%	75.34%
En2	With	0.16	0.20	0.26	69.63%	91.75%	97.27%	100%
	Without	0.50	0.56	0.66	7.69%	15.49%	47.15%	100%
	With raw	0.73	1.04	1.81	2.54%	12.06%	25.03%	67.19%
En3	With	0.15	0.22	0.45	70.42%	79.91%	90.22%	100%
	Without	0.60	0.68	0.98	7.95%	15.57%	30.40%	80.67%
	With raw	1.19	1.43	1.59	2.51%	2.51%	5.22%	70.12%

**Table 5:** Percentile of error reduction using corrected UWB ranging.

En1		En2		En3	
Mean	Sth	Mean	Sth	Mean	Sth
65.65%	56.67%	64.50%	46.13%	65.54%	41.09%

**Table 6:** Update time under different sample number.

Sample Number	1	10	50	100	150	200
Update time (ms)	<1	1.90	10.5	20.3	29.6	41.8

## Data Availability

The data presented in this study are available on request from the corresponding author.
